# New Diagnostic and Therapeutic Approaches for Preventing the Progression of Diabetic Retinopathy

**DOI:** 10.1155/2016/1753584

**Published:** 2015-12-31

**Authors:** Young Gun Park, Young-Jung Roh

**Affiliations:** Department of Ophthalmology and Visual Science, Catholic University of Korea, No. 62 Yeouido-dong, Yeongdeungpo-gu, Seoul 07345, Republic of Korea

## Abstract

Diabetic retinopathy (DR) is a severe sight-threatening complication of diabetes mellitus. Retinal laser photocoagulation, antivascular endothelial growth factors, steroid therapy, and pars plana vitrectomy are now used extensively to treat advanced stages of diabetic retinopathy. Currently, diagnostic devices like ultrawide field fundus fluorescein angiography and the improvement of optical coherence tomography have provided quicker and more precise diagnosis of early diabetic retinopathy. Thus, treatment protocols have been modified accordingly. Various types of lasers, including the subthreshold micropulse laser and RPE-targeting laser, and selective targeted photocoagulation may be future alternatives to conventional retinal photocoagulation, with fewer complications. The new developed intravitreal medications and implants have provided more therapeutic options, with promising results.

## 1. Introduction

Diabetic retinopathy (DR) is a sight-threatening complication of diabetes and is known to be the leading cause of blindness [1]. Twenty-eight million people in the United States have type 2 diabetes and more than 350 million people worldwide [2]. Typical ocular complications range from impaired visual acuity due to diabetic retinopathy and premature cataracts all the way to blindness or loss of an eye.

DR is characterized by gradual progressive retinal vasculopathy, leading to endothelial cell dysfunction, breakdown of the blood-retinal barrier, ischemia-induced retinal neovascularization, and expansion of the extracellular matrix, resulting in the outgrowth of fibrovascular tissue at the vitreoretinal interface [3]. In addition, recent studies indicate that chronic low-grade inflammation is involved in the pathogenesis of DR [4].

Diabetic retinopathy can be clinically classified into two stages: early stages like nonproliferative diabetic retinopathy (NPDR) and late stages like PDR [5]. Arresting of NPDR at an early level would be necessary to reduce the risk of severe visual loss. However, current treatments target late stages of DR when vision has already been significantly affected, so there is a need to stop the progression of DR earlier. Moreover, most of the treatments for advanced stages, such as conventional laser therapy, intravitreal anti-VEGF or corticosteroid injections, and vitreoretinal surgery, are expensive and invasive and have serious complications.

Earlier detection and timely treatment of sight-threatening DR have reduced the incidence and progression of visual loss [6, 7]. A multidisciplinary approach is needed to design new effective prevention strategies for the early stages of DR.

Although treatment controlling systemic risk factors including hyperglycemia and hypertension is crucial to preventing and arresting DR, here we focused on local rather than systemic treatment. In this paper, we provide an outline of current trends to treat and diagnose diabetic retinopathy in the ophthalmic field.

## 2. Current Ophthalmic Therapeutic Options

Current treatments target the later stage of DR, but it would be highly desirable to prevent the onset of the disease or arrest its progression at a stage before the appearance of overt microvascular pathology. Present ocular treatment revolves around four major strategies: retinal laser photocoagulation, anti-VEGF drugs, steroids, and surgical intervention (Table 1).

### 2.1. Laser Photocoagulation

Laser photocoagulation is the main treatment for established DR, and it is generally indicated in PDR or in clinically significant diabetic macular edema (CSME). This intervention prevents further deterioration of vision if applied sufficiently early in the progression of the disease but does not usually restore lost vision. The Diabetes Retinopathy Study (DRS) and Early Treatment Diabetes Retinopathy Study (ETDRS) groups developed guidelines for the laser treatment of diabetic retinopathy [8–10]. In DRS, panretinal photocoagulation (PRP) was shown to reduce the risk of severe visual loss by 60% in 2 years, especially in patients with PDR and high-risk characteristics [8]. Later, ETDRS suggested that patients with severe NPDR might also benefit from scatter photocoagulation as well [10].

The exact mechanism by which PRP aids in the regression of neovascularization is unclear. It appears to be a combined effect of multiple elements including facilitation of the transport of oxygen and nutrients into the retina from the choroid, the transport of metabolic waste out of the retina, reduction of retinal metabolic load, and reduced VEGF expression [11]. Other cytokines, such as heat shock proteins and transforming growth factor-*β*2, have been implicated in inflammatory responses in retinal photocoagulation as well [12, 13].

The utility of focal/grid laser therapy was studied in ETDRS, and the focal laser photocoagulation of microaneurysms and localized areas of leakage in patients with CSME was shown to reduce the incidence of moderate vision loss by 50% (from 24% to 12%) [9, 10]. Apart from the direct effect, by coagulating microaneurysms, the exact mechanisms of action of the focal laser treatment are still poorly understood. However, they are presumed to involve stimulation of RPE, closure of leaking microaneurysms, and the induction of endothelial cell proliferation. Moreover, increased oxygenation leads to constriction of arterioles, and alterations in various cytokines and growth factors play a role in reducing macular edema [14–17].

Although PRP is often used to successfully reduce the risk of severe vision loss from the progression of DR, significant visual side effects are also associated with the treatment, such as reduced peripheral vision with narrow visual field and decreased dark adaption due to loss of rod function. These interfered functions can affect the patients' driving ability and reduce color and contrast perception [18]. To minimize these side effects, less aggressive strategies, such as pattern scan laser (Pascal), subthreshold micropulse diode laser, retinal rejuvenation therapy (2RT), and selective retinal therapy (SRT), have been developed.

### 2.2. Intravitreal Anti-Vascular Endothelial Growth Factor (VEGF) Agents

Until the introduction of anti-VEGF medications, focal and grid laser photocoagulation was the standard treatment for CSME. However, center-involving diffuse diabetic macular edema limits functional prognosis with grid laser photocoagulation [9]. Thus, intravitreal anti-VEGF therapy has become the primary treatment compared to the grid laser photocoagulation. VEGF is known to cause leakage and retinal edema from the breakdown of the blood-retinal barrier [19]. Intravitreal injection of anti-VEGF medication allows high concentrations in the vitreous and avoidance of high systemic exposure.

Currently, four intravitreal anti-VEGF agents—pegaptanib (Macugen; Pfizer Inc., NY, USA), ranibizumab (Lucentis; Genentech Inc., San Francisco, CA, USA), bevacizumab (Avastin; Genentech Inc.), and aflibercept (Eylea; Regeneron Pharmaceuticals Inc., NY, USA)—have emerged as new treatments for the more advanced stages of DR. Recently, major randomized controlled clinical trials investigating the use of anti-VEGF medication for DME have been reported [20–22]. These trials have provided clear evidence that intraocular administration of anti-VEGF agents shows good results both in preserving and in improving vision for patients with DME.

In randomized controlled trials that used ranibizumab injections, a total of 345 patients with DME were enrolled and treated with 0.5 mg ranibizumab as adjunctive therapy to laser photocoagulation and/or monotherapy. Ranibizumab alone and ranibizumab plus laser significantly improved BCVA compared with laser monotherapy, showing +6.1 letters, +5.9 letters, and +0.8 letters at 12 months, respectively. Compared with laser treatment alone, ranibizumab groups showed higher proportion of gain of ≥10 and ≥15 letters (resp., 37.4% versus 15.5% and 22.6% versus 8.2%) [21].

Additionally, a recent study comparing the relative efficacy and safety of intravitreous ranibizumab, bevacizumab, and aflibercept in the treatment of DME concluded that they all improved vision, but the relative effect depended on baseline visual acuity. There was no apparent difference among the treatments when the initial visual acuity loss was mild. However, aflibercept was more effective at improving vision when initial visual acuity was worse [22].

Repeated injections of anti-VEGF medications are required in patients with center-involving diabetic macular edema. Unlike neovascular age-related macular degeneration, visual acuity in patients with DME could be maintained with tapering the injection frequency over time [23]. Recent studies indicated that the average number of treatment is reduced by the progression of time, with seven injections in the first year and four in the second one [21]. Currently, some experts favor combination focal/grid laser therapy and pharmacotherapy with intravitreal anti-VEGF agents in patients with CSME. Combination therapy was shown to reduce the frequency of injections needed to control edema [24].

Though intravitreal anti-VEGF therapy is generally safe with relatively low side effects of medication, it is an invasive procedure, which may lead to local complications such as endophthalmitis and retinal detachment. Moreover, anti-VEGF drugs, when delivered into the vitreous, can pass into the systemic circulation, which could potentially result in hypertension, proteinuria, increased cardiovascular events, and impaired wound healing. These systemic effects are rare but do occur [25].

### 2.3. Steroid Therapy

Intravitreal steroids have been reported to generate favorable results in the treatment of diabetic macular edema. Intravitreal triamcinolone acetonide (IVTA) has been used mainly for its anti-inflammatory activity. However, the incidence of complications with corticosteroid injection is high, with the most common being intraocular pressure elevation and cataract formation. For this reason, they are generally used in patients affected by laser-refractory DME, especially in pseudophakic eyes [26].

Moreover, a major limitation of IVTA is the recurrence of DME, which develops after a relatively short duration of action (not longer than 3 months) necessitating repeated applications of IVTA that carry risks and are inconvenient for patients. Recently, sustained-release devices have been introduced and can lengthen the intervals between retreatments [27].

Recent studies have demonstrated that the biodegradable dexamethasone (DEX) 0.7 mg sustained-release intravitreal implant (Ozurdex; Allergan Inc., Irvine, CA, USA) and a nonabsorbable implant containing 190 *μ*g fluocinolone acetonide (Retisert; Bausch and Lomb, Rochester, NY, USA) are promising new treatment options for patients with persistent DME [28, 29]. These implants provide sustained-release low-dose delivery, limiting the frequency of intravitreal injections and possibly reducing the costs associated with intravitreal anti-VEGF therapy. In a recent randomized clinical trial, it was shown that a DEX implant achieved similar rates of visual acuity improvement to bevacizumab for DME, with superior anatomic outcomes and fewer injections [30]. Despite such advantages, these devices are associated with cataract formation, increased intraocular pressure, and surgery to lower intraocular pressure [31, 32].

### 2.4. Surgical Treatment

In advanced cases of proliferative DR, with vitreous hemorrhage, tractional retinal detachment, and extensive fibrous membranes, pars plana vitrectomy should be performed [33]. The procedure is also used to remove the premacular posterior hyaloid from patients with persistent diffuse macular edema [34].

The Diabetic Retinopathy Clinical Research Network evaluated vitrectomy for DME associated with vitreomacular traction [35]. At 6 months, median OCT central subfield thickness decreased by 160 microns, and visual acuity improved by ≥10 letters in 38%. Factors associated with favorable outcomes after vitrectomy for DME were removal of an epiretinal membrane, removal of internal limiting membrane, and worse baseline visual acuity [36]. However, the need for internal limiting membrane peeling is still unclear.

The improvement in visual outcome achieved about 10 years ago mainly resulted from advances in vitreoretinal surgical instrumentation and technique enabling more effective removal of complex fibrovascular membranes [37]. Transconjunctival sutureless 23- or 25-gauge vitrectomy started to replace conventional 20-gauge technique and offers comparable safety and efficacy as well as reduced surgery times and faster rehabilitation of patients [38].

However, the visual outcome after vitrectomy remains unpredictable. In addition, significant postoperative complications may occur including cataract formation, recurrent vitreous hemorrhage, rhegmatogenous retinal detachment, and neovascular glaucoma [39].

## 3. Recent Developments in Early Diagnosis in Diabetic Retinopathy

Early detection and treatment of progressive retinal disease are another aspect of the management of DR. Important advances in diagnostic devices such as ultrawide field fundus fluorescein angiography (UWFA) and OCT allow early diagnosis of DR in recent years. Unlike fundus fluorescein angiography, peripheral capillary nonperfusion can also be visualized well using UWFA. OCT is another advance over the last decade that provides clear histology, including layers of retina and choroid. It provides retinal thickness to diagnose macular edema. Intraretinal cysts, vitreoretinal traction, epiretinal membranes, and other retinal macular pathologies of DR can also be diagnosed readily.

### 3.1. Ultrawide Field Fundus Fluorescein Angiography (UWFA)

**Figure 1:**
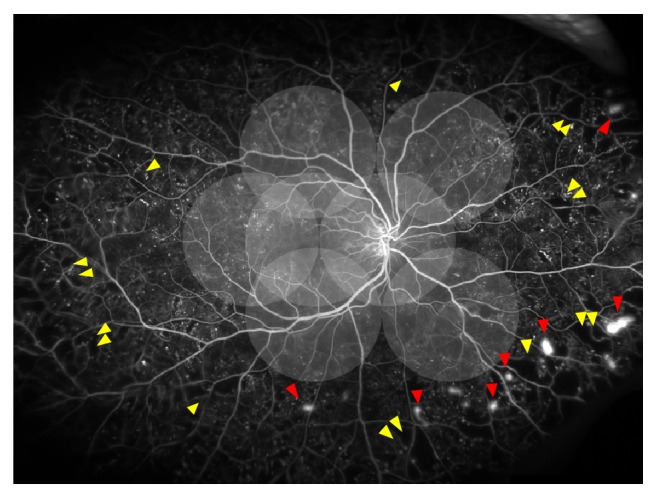
Diagram illustrating the extent of the seven standard ETDRS fields (the 75° view) on an ultrawide field fundus photo (the 200° view). Ultrawide field fundus angiography of a patient with proliferative diabetic retinopathy with extensive peripheral ischemia and extensive retinal neovascularization at the border between the perfused and nonperfused retinae.

Recently, the peripheral retina imaging has been focused in patients with DR. UWFA enables visualization of the peripheral retina up to 200° in a single frame and permits examining the peripheral capillary nonperfusion (Figure 1). One study reported that ultrawide field imaging (UWF) allowed visualization of retinal surface area 3.2 times more than the conventional ETDRS-protocol 7 standard field stereoscopic fundus photographs for the detection and management of diabetic retinopathy [40]. UWF has been increasingly used as the primary screening device for the diagnosis and monitoring of DR [41, 42]. Visualization of the peripheral retinal is improved with UWFA, and the device has provided clinical benefits and improved patient outcomes.

### 3.2. Optical Coherent Tomography (OCT)

OCT is a noninvasive imaging technique, and the images are presented with cross section of the retina in high resolution. Time-domain OCT (TD-OCT) shows an axial resolution of 8–10 *μ*m by using scan rates of 400 A-scans per second [43]. Unlike TD-OCT, spectral-domain OCT (SD-OCT) can improve the resolution of the image and visualize the choroid using both image averaging and enhanced depth imaging (EDI). Using an interferometer with a high-speed spectrometer, the interference spectrum measurement of SD-OCT system detects the light echoes simultaneously. An imaging speed of 20,000 to 52,000 A-scans/sec and an axial resolution of 5–7 *μ*m in tissue can be achieved with this technique. For diagnosing CSME, OCT is used for the gold standard test.

**Figure 2:**
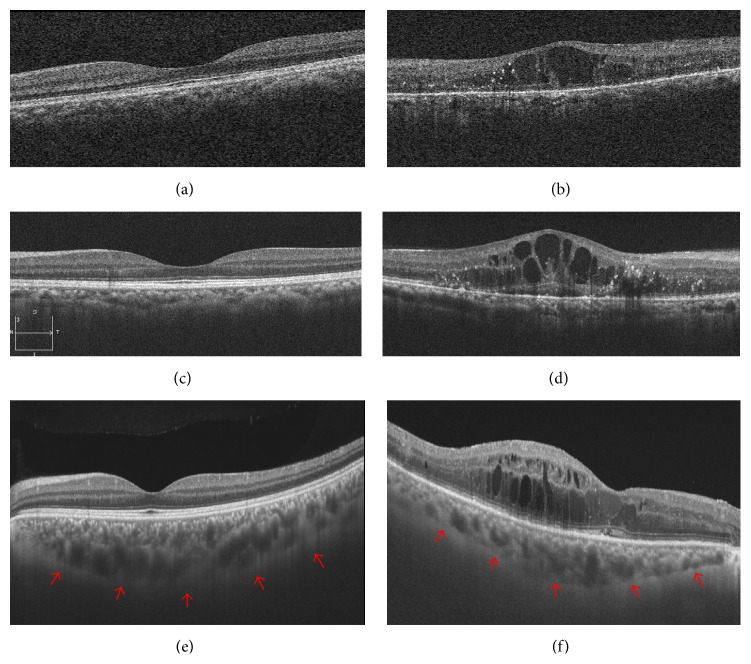
Optical coherence tomography (OCT) features of diabetic retinopathy in comparison with a normal eye. (a, b) OCT images in the normal and diabetic eyes obtained using the Cirrus HD-OCT system, respectively. (c, d) OCT images, respectively, in normal and diabetic eyes also obtained using the Cirrus HD-OCT system with the enhanced depth imaging (EDI) technique. (e, f) OCT images obtained using a swept-source OCT (SS-OCT). Note that the signal quality is improved markedly, and the vitreomacular interface and the choroid-sclera junction become clear. (e) The SS-OCT image of normal eye allows enhanced visualization of choroidal thickness (red arrows). (f) The image of a patient with DR showed reduced choroidal thickness.

**Figure 3:**
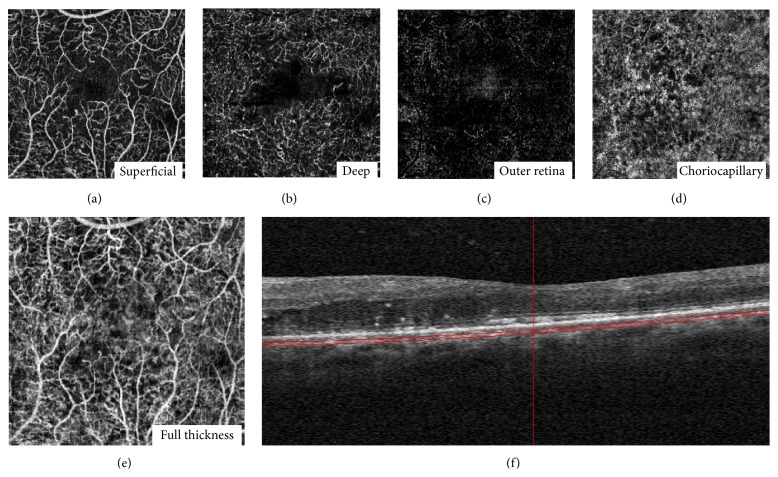
En face optical coherence tomography (OCT) angiography images of the layer segmentation and horizontal B-scan images in a patient with diabetic macular edema. (a) 3 × 3 mm OCT angiogram of the “superficial” inner retina. (b) 3 × 3 mm OCT angiogram of the “deep” inner retina. (c) 3 × 3 mm OCT angiogram of the outer retina shows absence of vasculature. The white represents noise. (d) 3 × 3 mm OCT angiogram of the choriocapillaris. There is black shadowing from the retinal vessels. (e) Full-thickness (internal limiting membrane to Bruch's membrane). (f) Highly sampled OCT b-scan image.

OCT can also demonstrate a number of microanatomical features in diabetic macular edema, as well as areas of subclinical macular edema [44]. Additionally, SD-OCT can be useful to evaluate choroidal thickness [45]. Swept-source OCT (SS-OCT) provides 3 *μ*m resolution in tissue using scan rates of 100,000 A-scans per second and improves the visualization of the choroid [46] (Figure 2). Recently, OCT-angiography using a split-spectrum amplitude-decorrelation angiography algorithm improved the visualization of the microcirculation in the retina and choroid [47]. En face imaging in OCT-angiography may be useful to evaluate the microvascular status of the DR in detail. It was shown that the extent of the nonperfused area differed between the superficial and deep plexuses of the macula region [48] (Figure 3).

A significantly thinner choroid was observed in type 2 diabetic patients without diabetic retinopathy or early stage DR. The patients with DME also showed reduced choroidal thickness [49, 50]. Thus, choroidal thickness could be used as a marker for the risk of developing DR. OCT-angiography is expected to be helpful for evaluating the choroidal vessels. Thus, further studies are needed to confirm the relationship between DR and choroidal thickness.

## 4. Current Preventive Trends in Treating Progression of DR

### 4.1. New Concepts in Laser Therapy

A new laser treatment was developed by modifying a diode laser in an effort to reduce unavoidable loss of the visual field from collateral damage of conventional continuous wave (CW) laser therapy. Compared with CW laser therapy, RPE-targeting lasers such as SDM, retinal rejuvenation therapy (2RT), and selective retina therapy (SRT) can cause less damage to the neural retina in DME using a short pulse duration like a microsecond or even a nanosecond [51–56]. Although the mechanism of SDM remains unknown, the laser may induce changes of cytokines in RPE and activate heat shock protein expression [13, 57]. Unlike the invisibility of SDM lesions on fundus photography and fluorescein angiography, laser spots of SRT can be visualized by fluorescein angiography due to RPE damage. However, these lesions disappeared on subsequent fluorescein angiography because the damaged area can be covered by the migration and proliferation of surrounding RPE cells [56, 58].

#### 4.1.1. Targeted Laser for Retinal Pigment Epithelium (RPE)

Subthreshold diode micropulse laser photocoagulation (SDM) uses a diode laser with a micropulse technique and lower fluence to achieve subthreshold burns to limit the laser burns to RPE [59]. Whereas conventional laser therapy uses continuous waves of energy delivery, micropulse mode divides a single energy delivery of the laser burn with cycles of on time and off time until the full duration is delivered without a visible burn endpoint [60]. Histopathological studies have demonstrated that the micropulse diode laser affects only RPE without damaging the outer retina [51]. Subsequently, several papers were published on the SDM in DME and found it to be equally effective as a conventional argon or threshold diode laser [51, 52]. However, there have been very few clinical studies on the RPE-targeting laser for treating progression of DR. A recent study demonstrated that pan retinal photocoagulation using SDM for DR ranging from severe nonproliferative to proliferative diabetic retinopathy showed a similar incidence of* de novo* vitreous hemorrhage and vitrectomy, compared with previous reports [61]. Additionally, SDM treatments could avoid the complications associated with conventional photocoagulation such as retinal damage, reduced vision, and reduced visual field. Because the RPE-targeting laser may induce different tissue responses depending on the degree of fundus pigmentation, our group is developing an algorithm of real time feed-back dosimetry of SRT system, which can help to deliver adequate energy for each individual person with different degrees of fundus pigmentation [56]. However, randomized long-term clinical trial is needed to confirm the efficacy of such RPE-targeting lasers for DR.

#### 4.1.2. Targeted Laser Therapy for Ischemic Retina Areas

Targeted laser therapy is intended to selectively treat ischemic retinal areas and adjacent intermediate areas showing angiographic leakage while minimizing some of the risks and complications of conventional PRP [62]. Although DRS and ETDRS groups suggested benefits from PRP, photocoagulation can trigger complications, such as diminished visual field, reduced contrast sensitivity, and impaired night vision.

Photocoagulation targeting only ischemic retinal areas has been performed widely in Japan. It has been reported that the selective photocoagulation group (PC group) for nonperfusion areas (NPA) in preproliferative diabetic retinopathy (PPDR) is more effective in preventing the progressing of DR compared with the conventional pan retinal photocoagulation group (non-PC group). Over 3 years, PDR developed in 18 (26%) of total number of the 69 patients. This incidence was significantly higher in the non-PC group (15/37 patients, 41%) than in the PC group (3/32, 9%) [63].

UWF allows identification of peripheral areas of nonperfusion and vascular leakage, and it can perform a role as a guide for targeted retinal photocoagulation (TRP) [62]. Silva et al. suggested that these peripheral lesions have implications for diagnosing more severe DR and peripheral pathology serves as a predictor of progression in diabetic retinopathy [64]. Although a clinical trial in Japan supported the idea that selective photocoagulation (S-PC) for nonperfusion areas in preproliferative DR is effective for preventing PDR development, a further long-term clinical trial is needed to confirm the efficacy of S-PC [63].

### 4.2. Anti-VEGF Agent for Diabetic Retinopathy

Although pan retinal photocoagulation was shown to reduce severe vision loss by 50% in the DRS report, no therapy reversed the progression of DR [5]. However, recent clinical studies of anti-VEGF for DME demonstrated that monthly ranibizumab for 36 months prevented the worsening of DR and induced severity scale reduction on the Early Treatment Diabetic Retinopathy Study. Patients with diabetic macular edema were randomized to monthly sham, 0.3 mg ranibizumab, or 0.5 mg ranibizumab intravitreal injections (*n* = 759). At 2 years, the percentage of participants with DR progression (worsening by 2 or 3 steps) was significantly reduced in ranibizumab-treated eyes compared with sham-treated eyes, and DR regression (improving by 2 or 3 steps) was significantly more likely. The cumulative probability of clinical progression of DR at 2 years was 33.8% of sham-treated eyes compared with 11.2% to 11.5% of ranibizumab-treated eyes. They demonstrated that intravitreal ranibizumab reduced the risk of DR progression and early intervention is important to reduce the DR severity level [19, 65]. This result supports the idea that anti-VEGF agents could be useful to control the progression of DR.

### 4.3. Intravitreal Corticosteroids

There is some basis for considering the question of whether corticosteroids could reduce the risk of progression of retinopathy, including development of proliferative diabetic retinopathy. Corticosteroids have been shown experimentally to downregulate VEGF production, reduce breakdown of the blood-retinal barrier, and possibly have antiangiogenic properties [66]. Moreover, intravitreal triamcinolone acetonide has been used in the prevention of retinal neovascularization in various studies [67].

Recently, several studies of intravitreal corticosteroids for DME showed the regression of DR [68, 69]. For example, patients with diabetic retinopathy were assigned randomly to laser or intravitreal triamcinolone acetonide (1 mg or 4 mg). After 2 years, the cumulative probability of progression of retinopathy was 31% (laser), 29% (1 mg), and 21% (4 mg), compared with laser group, *P* = 0.65 in the 1 mg group and 0.005 in the 4 mg group. These differences appeared to be sustained at 3 years [69]. As a result, intravitreal triamcinolone acetonide (4 mg) appeared to reduce the risk of progression of diabetic retinopathy.

However, use of this corticosteroid treatment just to reduce the progression of DR is not useful because of the possible complications such as glaucoma and cataracts and the need for reinjection due to short duration of action. If new steroid implants avoiding or lowering these complications are developed, corticosteroids could be another treatment option for controlling the progression of DR.

## 5. Conclusions

Improvements in diabetes care and management have been crucial in lowering the incidence and severity of DR. Nevertheless, the effectiveness of current treatments for DR is limited, and they are currently indicated at advanced stages of the disease. Thus, a multidisciplinary approach and novel strategies to detect, prevent, and treat DR in the early stages are needed. Several therapeutic strategies in the early stages of DR are being evaluated. However, when the early stages of DR are the therapeutic target, it would be recommended that less aggressive and more prudent treatments should be used, avoiding serious adverse effects.

New technologies in both retinal imaging and functional assessments, such as UWFA or OCT, will allow the detection of early changes and designing a personalized, noninvasive treatment. These efforts will be effective in reducing the burden and improving the clinical outcome of this potentially devastating complication of diabetes.

## Figures and Tables

**Table 1:** Summary of current ophthalmic therapeutic options for diabetic macular edema.

Category	Previous treatment options	New treatments options	Benefits of new treatments
Laser photocoagulation	Pan retinal photocoagulation Focal photocoagulation	Pattern scan laser (Pascal) Subthreshold diode micropulse laser (SDM) Retinal rejuvenation therapy (2RT) Selective retina therapy (SRT)	It reduces laser-induced side effects (constriction of visual fields, reduced dark adaption, and reduced color and contrast perception)

Anti-VEGF agent	Pegaptanib (Macugen) Ranibizumab (Lucentis) Bevacizumab (Avastin) Aflibercept (Eylea)	Anti-VEGF agents plus focal/grid laser therapy	(i) Intravitreal anti-VEGF therapy is generally safer (ii) Visual acuity could be maintained with tapering the injection frequency over time

Steroid	Intravitreal triamcinolone acetonide	Dexamethasone sustained-release intravitreal implant (Ozurdex) Fluocinolone acetonide implant (Retisert)	(i) It Reduces the frequency of intravitreal anti-VEGF injections (ii) It is less associated with cataract formation and increased intraocular pressure than the previous steroid agents

Surgical treatment	Conventional 20-gauge vitrectomy	Transconjunctival sutureless 23- or 25-gauge vitrectomy	It reduced surgery times and makes rehabilitation of patients faster

## References

[1] Thomas R. L., Dunstan F., Luzio S. D. (2012). Incidence of diabetic retinopathy in people with type 2 diabetes mellitus attending the diabetic retinopathy screening service for Wales: retrospective analysis. *British Medical Journal*.

[2] Shaw J. E., Sicree R. A., Zimmet P. Z. (2010). Global estimates of the prevalence of diabetes for 2010 and 2030. *Diabetes Research and Clinical Practice*.

[3] Shin E. S., Sorenson C. M., Sheibani N. (2014). Diabetes and retinal vascular dysfunction. *Journal of Ophthalmic & Vision Research*.

[4] El-Asrar A. M. A. (2012). Role of inflammation in the pathogenesis of diabetic retinopathy. *Middle East African Journal of Ophthalmology*.

[5] The Diabetic Retinopathy Study Research Group (1976). Preliminary report on effects of photocoagulation therapy. *American Journal of Ophthalmology*.

[6] Stefánsson E., Bek T., Porta M., Larsen N., Kristinsson J. K., Agardh E. (2000). Screening and prevention of diabetic blindness. *Acta Ophthalmologica Scandinavica*.

[7] Cheung N., Mitchell P., Wong T. Y. (2010). Diabetic retinopathy. *The Lancet*.

[8] The Diabetic Retinopathy Study Research Group (1978). Photocoagulation treatment of proliferative diabetic retinopathy: the second report of diabetic retinopathy study findings. *Ophthalmology*.

[9] (1991). Early photocoagulation for diabetic retinopathy—ETDRS report number 9. Early Treatment Diabetic Retinopathy Study Research Group. *Ophthalmology*.

[10] Early Treatment Diabetic Retinopathy Study Research Group (1987). Treatment techniques and clinical guidelines for photocoagulation of diabetic macular edema. Early Treatment Diabetic Retinopathy Study Report Number 2. *Ophthalmology*.

[11] Blumenkranz M. S. (2014). The evolution of laser therapy in ophthalmology: a perspective on the interactions between photons, patients, physicians, and physicists: the LXX Edward Jackson Memorial Lecture. *American Journal of Ophthalmology*.

[12] Matsumoto M., Yoshimura N., Honda Y. (1994). Increased production of transforming growth factor-*β*2 from cultured human retinal pigment epithelial cells by photocoagulation. *Investigative Ophthalmology and Visual Science*.

[13] Sramek C., Mackanos M., Spitler R. (2011). Non-damaging retinal phototherapy: dynamic range of heat shock protein expression. *Investigative Ophthalmology and Visual Science*.

[14] Bhagat N., Grigorian R. A., Tutela A., Zarbin M. A. (2009). Diabetic macular edema: pathogenesis and treatment. *Survey of Ophthalmology*.

[15] Arnarsson Á., Stefánsson E. (2000). Laser treatment and the mechanism of edema reduction in branch retinal vein occlusion. *Investigative Ophthalmology and Visual Science*.

[16] Ogata N., Ando A., Uyama M., Matsumura M. (2001). Expression of cytokines and transcription factors in photocoagulated human retinal pigment epithelial cells. *Graefe's Archive for Clinical and Experimental Ophthalmology*.

[17] Ogata N., Tombran-Tink J., Jo N., Mrazek D., Matsumura M. (2001). Upregulation of pigment epithelium-derived factor after laser photocoagulation. *American Journal of Ophthalmology*.

[18] Fong D. S., Girach A., Boney A. (2007). Visual side effects of successful scatter laser photocoagulation surgery for proliferative diabetic retinopathy: a literature review. *Retina*.

[19] Qaum T., Xu Q., Joussen A. M. (2001). VEGF-initiated blood-retinal barrier breakdown in early diabetes. *Investigative Ophthalmology and Visual Science*.

[20] Massin P., Bandello F., Garweg J. G. (2010). Safety and efficacy of ranibizumab in diabetic macular edema (RESOLVE study): a 12-month, randomized, controlled, double-masked, multicenter phase II study. *Diabetes Care*.

[21] Mitchell P., Bandello F., Schmidt-Erfurth U. (2011). The RESTORE study: ranibizumab monotherapy or combined with laser versus laser monotherapy for diabetic macular edema. *Ophthalmology*.

[22] The Diabetic Retinopathy Clinical Research Network (2015). Aflibercept, bevacizumab, or ranibizumab for diabetic macular edema. *The New England Journal of Medicine*.

[23] Elman M. J., Qin H., Aiello L. P. (2012). Intravitreal ranibizumab for diabetic macular edema with prompt versus deferred laser treatment: three-year randomized trial results. *Ophthalmology*.

[24] Ford J. A., Elders A., Shyangdan D., Royle P., Waugh N. (2012). The relative clinical effectiveness of ranibizumab and bevacizumab in diabetic macular oedema: an indirect comparison in a systematic review. *British Medical Journal*.

[25] Costagliola C., Agnifili L., Arcidiacono B. (2012). Systemic thromboembolic adverse events in patients treated with intravitreal anti-VEGF drugs for neovascular age-related macular degeneration. *Expert Opinion on Biological Therapy*.

[26] Bandello F., Preziosa C., Querques G., Lattanzio R. (2014). Update of intravitreal steroids for the treatment of diabetic macular edema. *Ophthalmic Research*.

[27] Nentwich M. M., Ulbig M. W. (2012). The therapeutic potential of intraocular depot steroid systems: developments aimed at prolonging duration of efficacy. *Deutsches Arzteblatt International*.

[28] Messenger W. B., Beardsley R. M., Flaxel C. J. (2013). Fluocinolone acetonide intravitreal implant for the treatment of diabetic macular edema. *Drug Design, Development and Therapy*.

[29] Dutra Medeiros M., Postorino M., Navarro R., Garcia-Arumí J., Mateo C., Corcóstegui B. (2014). Dexamethasone intravitreal implant for treatment of patients with persistent diabetic macular edema. *Ophthalmologica*.

[30] Amoaku W. M., Saker S., Stewart E. A. (2015). A review of therapies for diabetic macular oedema and rationale for combination therapy. *Eye*.

[31] Guigou S., Pommier S., Meyer F. (2015). Efficacy and safety of intravitreal dexamethasone implant in patients with diabetic macular edema. *Ophthalmologica*.

[32] Pearson P. A., Comstock T. L., Ip M. (2011). Fluocinolone acetonide intravitreal implant for diabetic macular edema: a 3-year multicenter, randomized, controlled clinical trial. *Ophthalmology*.

[33] Mason J. O., Colagross C. T., Vail R. (2006). Diabetic vitrectomy: risks, prognosis, future trends. *Current Opinion in Ophthalmology*.

[34] Simunovic M. P., Hunyor A. P., Ho I.-V. (2014). Vitrectomy for diabetic macular edema: a systematic review and meta-analysis. *Canadian Journal of Ophthalmology*.

[35] Diabetic Retinopathy Clinical Research Network Writing Committee, Haller J. A., Qin H. (2010). Vitrectomy outcomes in eyes with diabetic macular edema and vitreomacular traction. *Ophthalmology*.

[36] Flaxel C. J., Edwards A. R., Aiello L. P. (2010). Factors associated with visual acuity outcomes after vitrectomy for diabetic macular edema: diabetic retinopathy clinical research network. *Retina*.

[37] Arumi J. G., Boixadera A., Martinez-Castillo V., Corcóstegui B. (2009). Transconjunctival sutureless 23-gauge vitrectomy for diabetic retinopathy. Review. *Current Diabetes Reviews*.

[38] Park D. H., Shin J. P., Kim S. Y. (2010). Comparison of clinical outcomes between 23-gauge and 20-gauge vitrectomy in patients with proliferative diabetic retinopathy. *Retina*.

[39] Newman D. K. (2010). Surgical management of the late complications of proliferative diabetic retinopathy. *Eye*.

[40] Kaines A., Oliver S., Reddy S., Schwartz S. D. (2009). Ultrawide angle angiography for the detection and management of diabetic retinopathy. *International Ophthalmology Clinics*.

[41] Manivannan A., Plskova J., Farrow A., Mckay S., Sharp P. F., Forrester J. V. (2005). Ultra-wide-field fluorescein angiography of the ocular fundus. *American Journal of Ophthalmology*.

[42] Wessel M. M., Aaker G. D., Parlitsis G., Cho M., D'Amico D. J., Kiss S. (2012). Ultra-wide-field angiography improves the detection and classification of diabetic retinopathy. *Retina*.

[43] Sull A. C., Vuong L. N., Price L. L. (2010). Comparison of spectral/fourier domain optical coherence tomography instruments for assessment of normal macular thickness. *Retina*.

[44] Sikorski B. L., Malukiewicz G., Stafiej J., Lesiewska-Junk H., Raczynska D. (2013). The diagnostic function of OCT in diabetic maculopathy. *Mediators of Inflammation*.

[45] Spaide R. F., Koizumi H., Pozonni M. C. (2008). Enhanced depth imaging spectral-domain optical coherence tomography. *American Journal of Ophthalmology*.

[46] Unterhuber A., Považay B., Hermann B., Sattmann H., Chavez-Pirson A., Drexler W. (2005). In vivo retinal optical coherence tomography at 1040 nm—enhanced penetration into the choroid. *Optics Express*.

[47] Jia Y., Tan O., Tokayer J. (2012). Split-spectrum amplitude-decorrelation angiography with optical coherence tomography. *Optics Express*.

[48] Ishibazawa A., Nagaoka T., Takahashi A. (2015). Optical coherence tomography angiography in diabetic retinopathy: a prospective pilot study. *American Journal of Ophthalmology*.

[49] Gerendas B. S., Waldstein S. M., Simader C. (2014). Three-dimensional automated choroidal volume assessment on standard spectral-domain optical coherence tomography and correlation with the level of diabetic macular edema. *American Journal of Ophthalmology*.

[50] Regatieri C. V., Branchini L., Carmody J., Fujimoto J. G., Duker J. S. (2012). Choroidal thickness in patients with diabetic retinopathy analyzed by spectral-domain optical coherence tomography. *Retina*.

[51] Luttrull J. K., Dorin G. (2012). Subthreshold diode micropulse laser photocoagulation (SDM) as invisible retinal phototherapy for diabetic macular edema: a review. *Current Diabetes Reviews*.

[52] Luttrull J. K., Musch D. C., Mainster M. A. (2005). Subthreshold diode micropulse photocoagulation for the treatment of clinically significant diabetic macular oedema. *British Journal of Ophthalmology*.

[53] Luttrull J. K., Sinclair S. H. (2014). Safety of transfoveal subthreshold diode micropulse laser for fovea-involving diabetic macular edema in eyes with good visual acuity. *Retina*.

[54] Pelosini L., Hamilton R., Mohamed M., Hamilton A. P., Marshall J. (2013). Retina rejuvenation therapy for diabetic macular EDEMA: a pilot study. *Retina*.

[55] Roider J., Liew S. H. M., Klatt C. (2010). Selective retina therapy (SRT) for clinically significant diabetic macular edema. *Graefe's Archive for Clinical and Experimental Ophthalmology*.

[56] Park Y.-G., Seifert E., Roh Y. J., Theisen-Kunde D., Kang S., Brinkmann R. (2014). Tissue response of selective retina therapy by means of a feedback-controlled energy ramping mode. *Clinical and Experimental Ophthalmology*.

[57] Gao X., Xing D. (2009). Molecular mechanisms of cell proliferation induced by low power laser irradiation. *Journal of Biomedical Science*.

[58] Roider J., Michaud N. A., Flotte T. J., Birngruber R. (1992). Response of the retinal pigment epithelium to selective photocoagulation. *Archives of Ophthalmology*.

[59] Sivaprasad S., Elagouz M., McHugh D., Shona O., Dorin G. (2010). Micropulsed diode laser therapy: evolution and clinical applications. *Survey of Ophthalmology*.

[60] Dorin G. (2003). Subthreshold and micropulse diode laser photocoagulation. *Seminars in Ophthalmology*.

[61] Luttrull J. K., Musch D. C., Spink C. A. (2008). Subthreshold diode micropulse panretinal photocoagulation for proliferative diabetic retinopathy. *Eye*.

[62] Reddy S., Hu A., Schwartz S. D. (2009). Ultra wide field fluorescein angiography guided Targeted Retinal Photocoagulation (TRP). *Seminars in Ophthalmology*.

[63] The Japanese Society of Ophthalmic Diabetology (2012). Multicenter randomized clinical trial of retinal photocoagulation for preproliferative diabetic retinopathy. *Japanese Journal of Ophthalmology*.

[64] Silva P. S., Cavallerano J. D., Sun J. K., Soliman A. Z., Aiello L. M., Aiello L. P. (2013). Peripheral lesions identified by mydriatic ultrawide field imaging: distribution and potential impact on diabetic retinopathy severity. *Ophthalmology*.

[65] Ip M. S., Domalpally A., Sun J. K., Ehrlich J. S. (2015). Long-term effects of therapy with ranibizumab on diabetic retinopathy severity and baseline risk factors for worsening retinopathy. *Ophthalmology*.

[66] Diaz-Flores L., Gitoerrez R., Varela H. (1994). Angiogenesis: an update. *Histology and Histopathology*.

[67] Antoszyk A. N., Gottlieb J. L., Machemer R., Hatchell D. L. (1993). The effects of intravitreal triamcinolone acetonide on experimental pre-retinal neovascularization. *Graefe's Archive for Clinical and Experimental Ophthalmology*.

[68] Elman M. J., Aiello L. P., Beck R. W. (2010). Randomized trial evaluating ranibizumab plus prompt or deferred laser or triamcinolone plus prompt laser for diabetic macular edema. *Ophthalmology*.

[69] Bressler N. M., Edwards A. R., Beck R. W. (2009). Exploratory analysis of diabetic retinopathy progression through 3 years in a randomized clinical trial that compares intravitreal triamcinolone acetonide with focal/grid photocoagulation. *Archives of Ophthalmology*.

